# Quantitative assessment of the association between AXIN2 rs2240308 polymorphism and cancer risk

**DOI:** 10.1038/srep10111

**Published:** 2015-05-14

**Authors:** Juan Gong, Yuan Jiang, Ningbo Hao, Bo Zhu, Yongsheng Li

**Affiliations:** 1 Institute of Cancer, Xinqiao Hospital, Third Military Medical University, Chongqing 400037, China; 2 Department of Gastroenterology, Xinqiao Hospital, Third Military Medical University, Chongqing 400037, China

## Abstract

Axin2 is involved in the regulation of Wnt/β-catenin pathway and implicated in cancer development and progression. The association between *AXIN2* rs2240308 polymorphism and cancer risk has been examined in several case-control studies, but the conclusions were conflicting. Here we performed a meta-analysis to evaluate the role of rs2240308 in cancer risk. A total of 8 studies were included in this meta-analysis (1559 cancer cases and 1503 controls). The pooled odds ratios (OR) and the 95% confidence intervals (CIs) were assessed to evaluate the association of the *AXIN2* rs2240308 polymorphism with a susceptibility to cancer. A significantly decreased overall cancer risk was observed in the homozygous (TT *vs.* CC), heterozygous (CT *vs.* CC), dominant (CT+TT *vs.* CC) and allelic (T *vs.* C) models (*P* < 0.005), rather than that in the recessive (TT *vs.* CT+CC) model (*P* = 0.092)*. AXIN2* polymorphism rs2240308 was also associated with decreased cancer risk under all five models in lung cancer. However, *AXIN2* rs2240308 polymorphism was not associated with cancer risk under any above model in Turkish population and under homozygous, heterozygous, recessive models in Japanese population. These findings indicate that *AXIN2* rs2240308 polymorphism significantly and race-specifically correlates with decreased cancer risk.

The pathogenesis of cancer is complicated and has not been completely elucidated. The genetic factors are important intrinsic factors that play critical roles in tumorigenesis^1^,^2^. Abundant evidences indicate that single nucleotide polymorphisms (SNPs) of genes involve in the malignancy^3^,^4^. Therefore, identification of key genetic factors related to cancer risk is important for developing efficient strategies for cancer prediction and therapy.

The Wnt signaling pathway was primarily identified for its role in cancer development^5^. Wnt signaling pathway induces the expression of tumor-related genes and promotes cancer progression through promoting the stabilization of cytoplasmic β-catenin^6^. β-catenin is regulated by axis inhibition protein 1 (Axin1) and its homologue Axin2. Axins interact with adenomatous polyposis coli (APC) and glycogen synthase kinase-3β (GSK-3β) and function as tumor suppressors^7^. The *AXIN2* gene is located at human chromosome 17q24, which consists of 10 exons encoding an 843-amino acid protein^8^. The mutation of the *AXIN2* gene and the loss of heterozygosity in the genomic locus have been observed in some cancers, such as hepatocellular carcinoma, ovarian cancer and colorectal carcinoma^9^.

Several SNPs have been identified in *AXIN2* coding region, including rs2240308 (exon1), rs9915936 (exon5), rs1133683 (exon5), and rs4072245 (intron7). Among these *AXIN2* SNPs, rs2240308 (exon1, 148C/T) is the most studied SNP and is closely related to cancer risk. The associations between rs2240308 and the risk of multiple solid cancers, such as lung cancer, colorectal cancer, head and neck cancer, astrocytoma, prostate cancer and ovarian cancer have been examined^9^,^10^,^11^,^12^,^13^,^14^,^15^,^16^. However, the results were inconsistent. In view of the importance of Axin2 in tumorigenesis, the present study systematically assessed the association between *AXIN2* rs2240308 (exon1, 148C/T) polymorphism and cancer risk through a meta-analysis.

## Results

### The main characteristics of included studies

**Figure 1:**
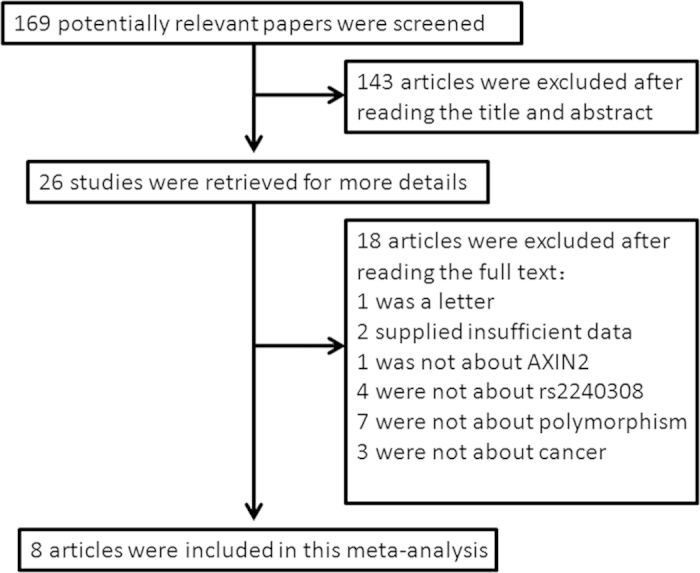
Flow chart of literature search and data extraction.

As shown in Fig. 1, totally 169 published papers were obtained with a combination of search terms as “*AXIN2* or axin 2”, “polymorphism or variant or SNP”, and “cancer or tumor or carcinoma”. 143 references were excluded by reading the title and abstract. After scanning the full text, 8 articles were included in this meta-analysis. 1559 cancer cases and 1503 controls were included in these articles. The 1559 cancer cases included lung cancer, colorectal cancer, head and neck cancer, astrocytoma, prostate cancer and ovarian cancer^9^,^10^,^11^,^12^,^13^,^14^,^15^,^16^. The populations included in these studies were Chinese, Japanese, Turkish, Iranian and Polish. All the included studies were consistent with the inclusion and exclusion criteria as indicated in detail in Methods. The genotype in control populations was conformed to Hardy–Weinberg equilibrium (HWE). The characteristics of included studies were shown in Supplementary Table 1. Distributions of genotypes and allele frequencies of *AXIN2* rs2240308 in cases and controls were indicated in Supplementary Table 2.

### Quantitative data synthesis

**Figure 2:**
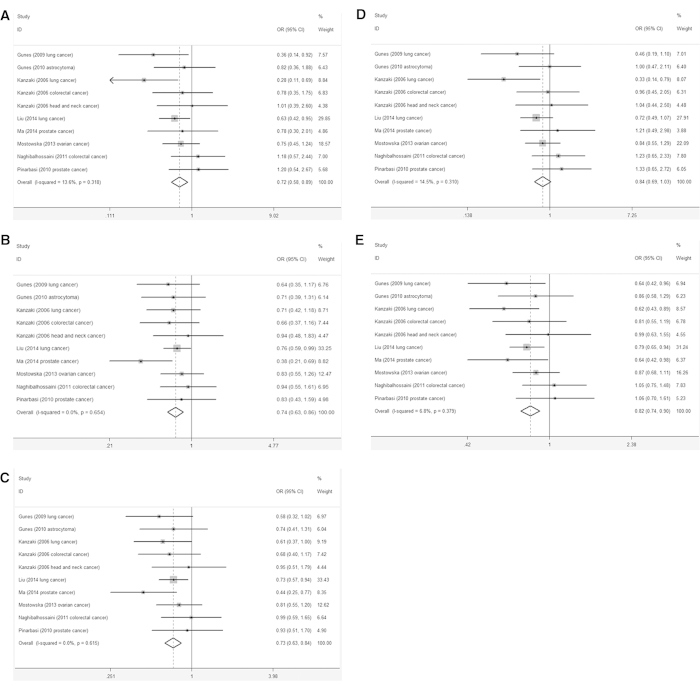
Forest plot of the overall cancer risk associated with the AXIN2 rs2240308 polymorphism. (**A**) TT *vs.* CC, (B) CT *vs.* CC, (**C**) CT+TT *vs.* CC, (**D**)TT *vs.* CT+CC, (**E**) T *vs.* C.

The heterogeneity among the selected studies was evaluated by Chi-squared test, *P* value < 0.05 means the heterogeneity was observed. If the heterogeneity among the selected studies was observed, the random-effects model would be applied to analysis the odds ratios (ORs) and their respective 95% confidence intervals (CIs); otherwise the fixed-effects model was used^17^,^18^. Since the heterogeneity was not observed in homozygous (TT *vs.* CC), heterozygous (CT *vs.* CC), dominant (CT+TT *vs.* CC), allelic (T *vs.* C) and recessive (TT *vs.* CT+CC) models (all *P* values >0.05, Supplementary Table 3), the fixed-effects model was used in the analysis. The ORs and their respective 95% CIs were used to evaluate the association between *AXIN2* rs2240308 and cancer risk. The Z test was applied to test the statistical significance of the pooled OR value. A significantly decreased overall cancer risk was found in the homozygous (TT *vs.* CC:OR = 0.72, 95% CI: 0.58-0.89, *P* = 0.003), heterozygous (CT *vs.* CC: OR = 0.74, 95% CI: 0.63-0.86, *P* < 0.001), dominant (CT+TT *vs.* CC: OR = 0.73, 95% CI: 0.63-0.84, *P* < 0.001) and allelic (T *vs.* C: OR = 0.82, 95% CI: 0.74-0.90, *P* < 0.001) models, but the decreased overall cancer risk was not observed in the recessive (TT *vs.* CT+CC: OR = 0.84, 95% CI: 0.69-1.03, *P* = 0.092) model (Fig. 2).

**Figure 3:**
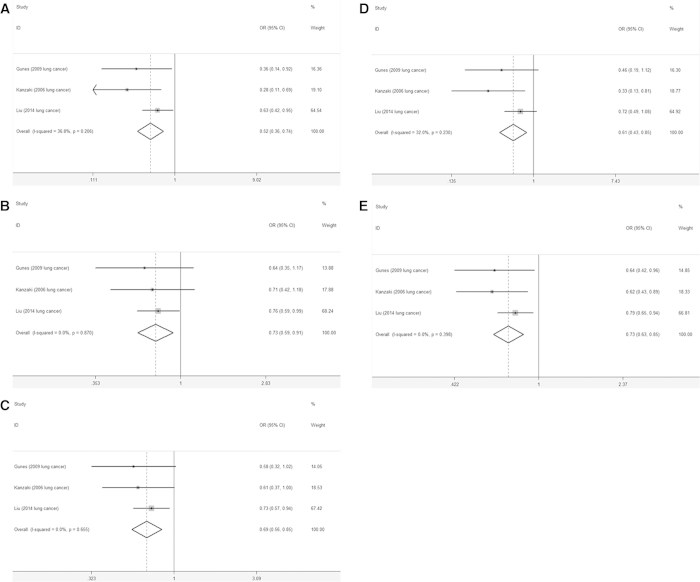
Forest plot of lung cancer risk associated with the AXIN2 rs2240308 polymorphism. (**A**) TT *vs.* CC, (**B**) CT *vs.* CC, (**C**) CT+TT *vs.* CC, (**D**)TT *vs.* CT+CC, (**E**) T vs. C.

The fixed-effects model was used in the analysis of lung cancer subgroup due to the absence of heterogeneity in all above models (Supplementary Table 3). Consistently, *AXIN2* rs2240308 was significantly associated with decreased lung cancer risk in these models, *i.e.*, the homozygous (TT *vs.* CC: OR = 0.52, 95% CI: 0.36-0.74, *P* < 0.001), heterozygous (CT *vs.* CC: OR = 0.73, 95% CI: 0.59-0.91, *P* = 0.005), dominant (CT+TT *vs.* CC: OR = 0.69, 95% CI: 0.56-0.85, *P* < 0.001), recessive (TT *vs.* CT+CC: OR = 0.61, 95% CI: 0.43-0.85, *P* = 0.003) and allelic (T *vs.* C: OR = 0.73, 95% CI:0.63-0.85, *P* < 0.001) models (Fig. 3).

**Figure 4:**
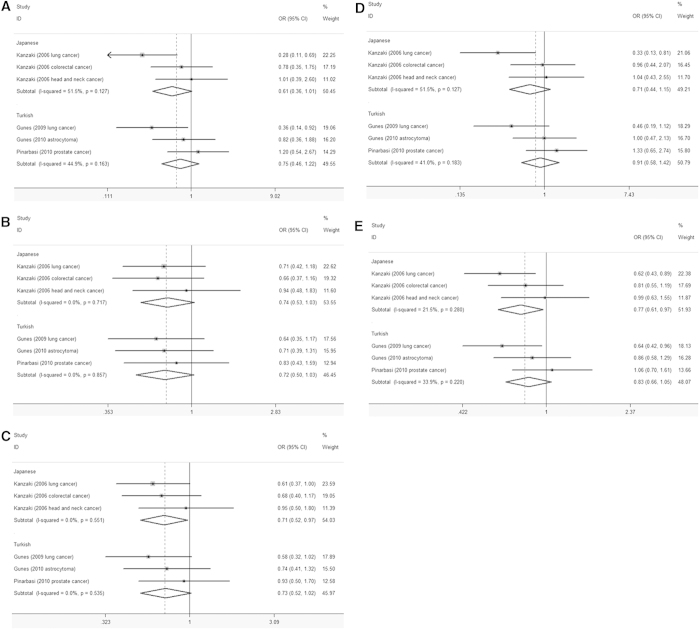
Forest plot of cancer risk of Japanese and Turkish associated with the AXIN2 rs2240308 polymorphism. (**A**) TT *vs.* CC, (**B**) CT *vs.* CC, (**C**) CT+TT *vs.* CC, (**D**) TT *vs.* CT+CC, (**E**) T *vs.* C.

Subgroup analysis based on population was also performed in this analysis. Heterogeneity was not observed in all five models (Supplementary Table 3) and thus the fixed-effects model was employed in this meta-analysis. As shown in Fig. 4, the association between *AXIN2* polymorphism rs2240308 and cancer risk was not observed in Turkish population under all these models, including the homozygous (TT *vs.* CC: OR = 0.75, 95% CI: 0.46-1.22, *P* = 0.250), heterozygous (CT *vs.* CC: OR = 0.72, 95% CI: 0.50-1.03, *P* = 0.069), dominant (CT+TT *vs.* CC: OR = 0.73, 95% CI: 0.52-1.02, *P* = 0.064), recessive (TT *vs.* CT+CC: OR = 0.91, 95% CI: 0.58-1.42, *P* = 0.669) and allelic (T *vs.* C: OR = 0.83, 95% CI: 0.66-1.05, *P* = 0.130) models. However, the *AXIN2* polymorphism rs2240308 was significantly associated with decreased cancer risk in Japanese under the dominant (CT+TT *vs.* CC: OR = 0.71, 95% CI: 0.52-1.97, *P* = 0.032) and allelic (T *vs.* C: OR = 0.77, 95% CI: 0.61-0.97, *P* = 0.024) models, but not in the homozygous (TT *vs.* CC: OR = 0.61, 95% CI: 0.36-1.01, *P* = 0.056), heterozygous (CT *vs.* CC: OR = 0.74, 95% CI: 0.53-1.03, *P* = 0.075) and recessive (TT *vs.* CT+CC: OR = 0.71, 95% CI: 0.44-1.15, *P* = 0.164) models.

### Sensitivity analyses and publication bias

**Figure 5:**
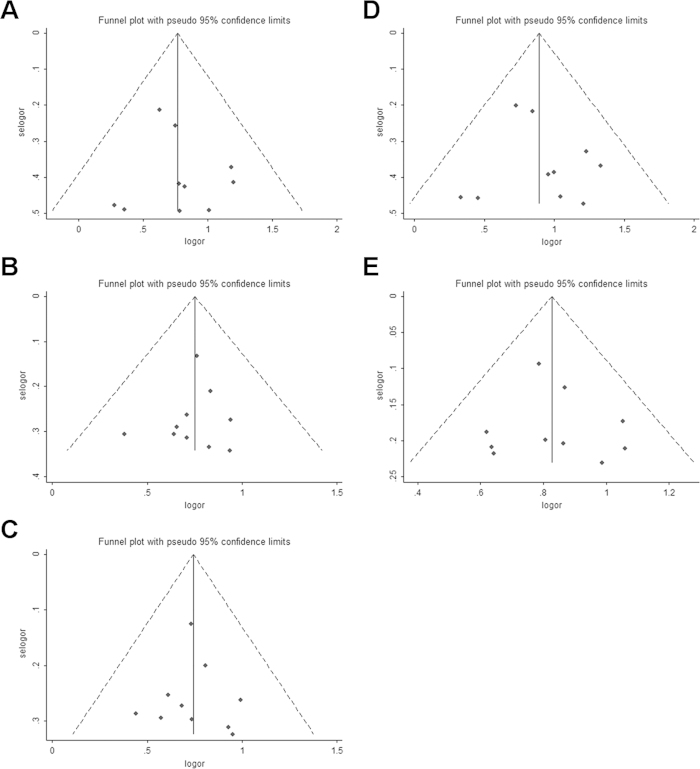
Funnel plot of overall cancer risk associated with the AXIN2 rs2240308 polymorphism for publication bias. (**A**) TT *vs.* CC, (**B**) CT *vs.* CC, (**C**) CT+TT *vs.* CC, (**D**)TT *vs.* CT+CC, (**E**) T *vs.* C.

To validate our results, we next performed sensitivity analysis. The corresponding pooled ORs were generally similar before or after single study was excluded each time or after random-effects model was used instead of the fixed-effects models. Egger’s test was performed in overall cancer risk analysis to assess the publication bias of the included studies. No publication bias was observed (*P* *>* 0.05). Moreover, the shape of funnel plots was nearly symmetrical for overall cancer risk under the homozygous, heterozygous, dominant, recessive and allelic models (Fig. 5). These results indicate that our conclusion in this meta-analysis was stable and credible.

## Discussion

Axin2 is a scaffold protein which is required for the phosphorylation of β-catenin. As a component of the Wnt pathway, the association between *AXIN2* polymorphism and carcinogenesis has been studied extensively, and a possible role for *AXIN2* polymorphism in cancer was suggested^9^,^10^,^11^,^12^,^13^,^14^,^15^,^16^. *AXIN2* has multiple SNPs including rs7210356, rs4791171, rs3923086 and rs2240308^12^,^19^. The rs2240308 polymorphism is a SNP in *AXIN2* exon1, which results in a serine to proline substitution mutation. The association between rs2240308 and cancer risk has been extensively investigated, but the conclusions were inconsistent. In this meta-analysis, we found that rs2240308 is significantly associated with decreased overall cancer risk under the homozygous (OR = 0.72), heterozygous (OR = 0.74), dominant (OR = 0.73) and allelic (OR = 0.82) models, rather than under the recessive model (OR = 0.84). In lung cancer, significant association between rs2240308 and decreased cancer risk was observed under the above five models. The conclusion was stable and credible as indicated by sensitivity analysis and publication bias analysis.

In contrast, significant decreased cancer risk was not observed under all five models in Turkish population. Nonetheless, *AXIN2* polymorphism rs2240308 was significantly associated with the cancer risk in Japanese population under dominant and allelic models but not under homozygous, heterozygous and recessive models, this result suggests that the association between *AXIN2* rs2240308 polymorphism and decreased cancer risk is race and model dependent.

It is noteworthy that this meta-analysis has its limitations. First, the included studies were published in English, while studies published in other languages were ignored. Second, a portion of the controls may have been exposed to unknown bias factors because they were hospital based. Third, the lack of individual-level data limited the further study for the interaction between the SNP and the metabolic traits. Nevertheless, the results of our meta-analysis are valid according to the analysis on the sensitivity and the significant publication bias which was evaluated through funnel plot and quantitative Egger’s test.

In conclusion, this meta-analysis indicated that the *AIXN2* rs2240308 polymorphism contributes to decreased overall cancer risk except for that under the recessive model. Although this association was not shared in Turkish population under the homozygous, heterozygous, dominant, allelic and recessive models or in Japanese population under the homozygous, heterozygous and recessive models, our findings highlight *AIXN2* rs2240308 polymorphism as a potential target for the control of cancer race-specifically.

## Methods

### Literature search and data extraction

The literature searches were performed by searching PubMed, MEDLINE and EMBASE (updated to February, 2015). The combination of search includes “*AXIN2* or axin 2”, “polymorphism or variant or SNP”, and “cancer or tumor or carcinoma”. Studies were eligible if they met the following criteria: (a) the association between *AXIN2* exon1 148 C/T (rs2240308) polymorphism and cancer risk was investigated; (b) all patients were diagnosed as cancer confirmed by pathological examination; (c) studies were published in English; (d) case–control studies with detailed either genotype or allele data estimating the ORs and 95% CIs and (e) the distribution of genotypes among controls were consistent with the HWE. In addition, criteria for exclusion of studies were: (a) case reports, family-based studies, abstracts, editorials and review articles; (b) overlapping data and (c) studies that reported neither genotype frequency nor allele frequency.

All of the studies were included or excluded according to above criteria. The accuracy of the extracted raw data was validated by two independent researchers (J.G. and Y.J.). Both researchers reached the same conclusion. The collected data included the first author, publication year, population, cancer type, the numbers of cases and controls, genotype distributions, matching criteria, control source, genotyping methods and HWE.

### Statistical analysis

We performed the analysis with Stata Statistical package 12.0 (Stata Corp LP, College Station, TX) and used the homozygous (TT *vs.* CC), heterozygous (CT *vs.* CC), dominant (CT+TT *vs.* CC), recessive (TT *vs.* CT+CC) and allelic (T *vs.* C) models in this meta-analysis. The association between the *AXIN2* rs2240308 polymorphism and the risk of cancer was determined by the ORs and their corresponding 95% CIs. The Z test was used to evaluate the statistical significance of the pooled OR value, and *P* < 0.05 was considered statistically significant. Heterogeneity was assessed by the Chi-squared and I-squared test, *P* < 0.05 for Chi-squared test was considered as heterogeneity among the studies, the ORs were determined with the random-effects model when *P* < 0.05, while *P* > 0.05 represented that the fixed-effects model was performed^17^,^18^. HWE in the controls was measured by Chi-squared test with the significance set at *P* < 0.05. For assessing the stability of the results, the sensitivity was assessed. Publication bias was analyzed by the use of funnel plot and Egger’s test^20^.

## Additional Information

**How to cite this article**: Gong, J. *et al*. Quantitative assessment of the association between AXIN2 rs2240308 polymorphism and cancer risk. *Sci. Rep.* **5**, 10111; doi: 10.1038/srep10111 (2015).

## Supplementary Material

Supplementary Information
