# Supplementary material for: Quantitative assessment of the association between AXIN2 rs2240308 polymorphism and cancer risk
Source: Sci Rep. 2015 May 14;5:10111. doi: 10.1038/srep10111 (PMC4431355; doi:10.1038/srep10111)
Supplement: Supplementary Information [file srep10111-s1.pdf]

Supplementary Information for:

Quantitative assessment of the association between AXIN2 rs2240308 polymorphism and cancer risk

Authors: Juan Gong[1,#], Yuan Jiang[1,#], Ningbo Hao[2], Bo Zhu[1,*], Yongsheng Li[1,*]

Affiliations:

[1]Institute of Cancer; [2]Department of Gastroenterology, Xinqiao Hospital, Third Military Medical University, Chongqing 400037, China

[#] These authors contributed equally to this work.

[*]Correspondence: Bo Zhu or Yongsheng Li, Institute of Cancer, Xinqiao Hospital, Third Military Medical University, Chongqing 400037, China. Email: b.davis.zhu@gmail.com (B.Z.) or yli@tmmu.edu.cn (Y.L.); Tel (Fax): 86-23-68774705 or 86-23-68755626.

Table.S1 Characteristics of studies included in meta-analysis

Study	Years	Populations	Cancer types	No.of Case/control	Matching Criteria	Control Source	Genotyping	HWE(P)
Kanzaki	2006	Japanese	Lung cancer<br>Colorectal cancer<br>Head and neck cancer	336/109	Smoking habit<br>Sex<br>Age	PB	PCR-RELP	0.8632
Gunes	2009	Turkish	Lung cancer	100/100	Sex<br>Age	PB	PCR	0.5008
Gunes	2010	Turkish	Astrocytoma	100/100	Sex<br>Age	HB	PCR	0.5008
Pinarbasi	2010	Turkish	prostate cancer	84/100	Age	HB	PCR	0.8825
Naghibalhossaini	2011	Iranian	Colorectal cancer	110/179	NA	NA	PCR-RFLP	0.0958
Mostowska	2013	Polish	Ovarian cancer	228/282	Sex<br>Age	HB	PCR-RFLP	0.5463
Ma	2014	Chinese	Prostate	103/100	Age<br>Geographic origin	PB	PCR	0.1531
Liu	2014	Chinese	Lung cancer	498/533	Sex<br>Age<br>Geographic origin	PB	PCR	0.4574

NA:not available; PB:population based; HB:hospital based; PCR-RFLP:PCR-restriction fragment length polymorphism;HWE(P) was tested by Chi-square test

Table.S2 Genotypes and allele frequencies of AXIN2 rs2240308(Exon 1 148 C/T) genes in cases and controls

			Case					Control					
Author	Year	Cancer type	CC	CT	TT	C	T	CC	CT	TT	C	T	Sample size
Kanzaki	2006	Lung cancer	81	71	8	233	87	42	52	15	136	82	160/109
Gunes	2009	Lung cancer	45	47	8	137	63	32	52	16	116	84	100/100
Liu	2014	Lung cancer	235	216	47	686	310	211	255	67	677	389	498/533
Kanzaki	2006	Colorectal cancer	54	44	15	152	74	42	52	15	136	82	113/109
Naghibalhossaini	2011	Colorectal cancer	34	57	19	125	95	55	98	26	208	150	110/179
Kanzaki	2006	Head and neck cancer	25	29	9	79	47	42	52	15	136	82	63/109
Gunes	2010	Astrocytoma	39	45	16	123	77	32	52	16	116	84	100/100
Pinarbasi	2010	Prostate cancer	30	35	19	95	73	34	48	18	116	84	84/100
Ma	2014	Prostate cancer	61	31	11	153	53	39	52	9	130	70	103/100
Mostowska	2013	Ovarian cancer	67	115	46	249	207	71	146	65	288	276	228/282

Table.S3 Heterogeneity assessment

Overall cancer risk associated with the AXIN2 rs2240308 polymorphism

	chi-squared test			I-squared test
	chi-squared	d.f.	p	I-squared(variation in OR attributable to heterogeneity)
TT vs. CC	10.41	9	0.318	13.60%
CT vs. CC	6.84	9	0.654	0.00%
CT+TT vs. CC	7.21	9	0.615	0.00%
TT vs. CT+CC	10.52	9	0.31	14.50%
T vs. C	9.66	9	0.379	6.80%

Lung cancer risk associated with the AXIN2 rs2240308 polymorphism

	chi-squared test			I-squared test
	chi-squared	d.f.	p	I-squared(variation in OR attributable to heterogeneity)
TT vs. CC	3.16	2	0.206	36.80%
CT vs. CC	0.28	2	0.87	0.00%
CT+TT vs. CC	0.85	2	0.655	0.00%
TT vs. CT+CC	2.94	2	0.23	32.00%
T vs. C	1.84	2	0.398	0.00%

Japanese cancer risk associated with the AXIN2 rs2240308 polymorphism

	chi-squared test			I-squared test
	chi-squared	d.f.	p	I-squared(variation in OR attributable to heterogeneity)
TT vs. CC	4.13	2	0.127	51.50%
CT vs. CC	0.67	2	0.717	0.00%
CT+TT vs. CC	1.19	2	0.551	0.00%
TT vs. CT+CC	4.12	2	0.127	51.50%
T vs. C	2.55	2	0.28	21.50%

Turkish cancer risk associated with the AXIN2 rs2240308 polymorphism

	chi-squared test			I-squared test
	chi-squared	d.f.	p	I-squared(variation in OR attributable to heterogeneity)
TT vs. CC	3.63	2	0.163	44.90%
CT vs. CC	0.31	2	0.857	0.00%
CT+TT vs. CC	1.25	2	0.535	0.00%
TT vs. CT+CC	3.39	2	0.183	41.00%
T vs. C	3.03	2	0.22	33.90%
